# Bis-three-way junction nanostructure and DNA machineries for ultrasensitive and specific detection of BCR/ABL fusion gene by chemiluminescence imaging

**DOI:** 10.1038/srep32370

**Published:** 2016-08-31

**Authors:** Yongjie Xu, Xintong Bian, Ye Sang, Yujian Li, Dandan Li, Wei Cheng, Yibing Yin, Huangxian Ju, Shijia Ding

**Affiliations:** 1 Key Laboratory of Clinical Laboratory Diagnostics (Ministry of Education), College of Laboratory Medicine, Chongqing Medical University, Chongqing 400016, China; 2 State Key Laboratory of Analytical Chemistry for Life Science, Department of Chemistry, Nanjing University, Nanjing 210093, China; 3 The Center for Clinical Molecular Medical detection, The First Affiliated Hospital of Chongqing Medical University, Chongqing 400016, China

## Abstract

A novel G-quadruplex DNAzyme-driven chemiluminescence (CL) imaging method has been developed for ultrasensitive and specific detection of BCR/ABL fusion gene based on bis-three-way junction (bis-3WJ) nanostructure and cascade DNA machineries. Bis-3WJ probes are designed logically to recognize BCR/ABL fusion gene, which forms the stable bis-3WJ nanostructure for the activation of polymerase/nicking enzyme machineries in cascade, resulting in synthesis of DNAzyme subunits. These DNAzyme subunits can form integrated DNAzyme by self-assembly to catalyze CL substrate, thus providing an amplified signal for the sensing events or outputs for AND logic operation. The imaging method achieved ultrasensitive detection of BCR/ABL fusion gene with a low detection limit down to 23 fM. And this method exhibited wide linear ranges over seven orders of magnitude and excellent discrimination ability toward target. In addition, an acceptable recovery was obtained in complex matrix. It is notable that this biosensing strategy possesses merits of homogenous, isothermal and label-free assay system. Therefore, these merits endow the developed imaging method with a potential tool for CML diagnosis.

Chronic myelogenous leukemia (CML), caused by BCR/ABL fusion gene, is a common malignancy of the pluripotent hematopoietic stem cell^1^,^2^. BCR/ABL fusion gene is the result of reciprocal translocation of chromosomes 9 and 22 (t [9; 22] [q^34^; q^11^]), leading to the generation of Philadelphia chromosome (Ph 1), which is the characteristic feature of the CML for existence in almost 95% patients^3^,^4^. At present, BCR/ABL fusion gene can serve as biomarker for monitoring disease transition from a chronic phase to acute stages and the presence of remaining leukemic cells during and after treatment in CML patients^5^. Therefore, it is of great significance to detect BCR/ABL fusion gene for early diagnosis and monitoring of the effectiveness of treatment.

Conventional methods for detecting BCR/ABL fusion gene mainly include real-time quantitative reverse transcription PCR^6^, flow cytometry^7^, chromosome analysis^8^, fluorescence *in situ* hybridization^9^, etc. However, these methods are often limited by technical complexity, costly instrumentations and low sensitivity. Thus, detection methods with merits of simplicity, high sensitivity and specificity are highly desired.

Recently, aiming at the improvement of analytical performance, great efforts are directed to develop biosensing strategies for BCR/ABL fusion gene detection as alternative methods. For example, different electrochemical biosensors have been developed for BCR/ABL fusion gene detection^10^,^11^. Despite these electrochemical biosensors are specific and cost-effective, they suffer from laborious probe immobilization and separation steps. Notably, chemiluminescence, as a most widely and efficient detection method in clinical diagnosis, can overcome the limitations by homogeneous assay system and render a convenient and potential tool for BCR/ABL fusion gene assay due to simplicity, high sensitivity and visual detection^12^,^13^. Thus, it is an attracting area to implement chemiluminescent biosensor in the determination of BCR/ABL fusion gene.

Self-assembly of nucleic acids provides a powerful and ideal approach for constructing molecular nanostructures and nanodevices by employing programmed DNA as a material^14^, including the development of DNA machines^15^,^16^,^17^, tailoring of DNA nanostructures^18^,^19^,^20^, etc. Up to now, self-assembly has been applied in many directions^21^, such as directed material assembly^22^, drug delivery^23^,^24^ and bioanalysis^25^,^26^. In bioanalysis, different biosensors have been developed for determination of a variety of analytes, including DNAzyme wires based fluorescent biosensors^27^, dendritic nanostructures based fluorescent biosensor^28^, tetrahedral DNA nanostructures based electrochemical biosensors^29^,^30^,^31^, three way junction^32^ and ferris wheel nanostructures^33^ based colorimetric biosensor and so on. These architectures provide a convenient solution to improve analytical performance by the merits of precise and automated spatial control, as well as simple and robust implementation in biosensing systems. In addition, these nanostructures can function as model structures for artificial switchable systems, molecular keypad-lock systems and amplified analysis systems^34^,^35^.

Inspired by these advantages of functional nanostructures, it is of great significance to explore potential nanostructures for the special recognition of target. To date, although DNA nanostructure play important roles in enhancing specificity, detection only based on these nanostructures without the assistance of amplifications is still limited by sensitivity because the ratio of target-to-signal is 1:1^36^. For increasing detection sensitivity, effective signal amplification strategies are highly required to transduce molecular recognition events. Thus, integration of functional nanostructure with potential signal amplifiers may offer a powerful tool for detecting BCR/ABL fusion gene on CL imaging platform.

In the present work, based on DNA nanotechnology and CL imaging platform, we develop a novel CL imaging method for ultrasensitive and specific detection of BCR/ABL fusion gene by utilizing bis-3WJ nanostructure as the result of AND logical recognition event, and cascade DNA machineries as signal amplifiers. The cascade DNA machineries ensure the enhanced output signals for the AND gate and the detection sensitivity. Also, the logic control of computational module corresponding to target responsive bis-3WJ nanostructure enhances the detection specificity. Importantly, this biosensing system possesses merits of homogenous, isothermal and label-free assay system, providing a convenient and powerful tool for BCR/ABL detection.

## Results and Discussion

### Principle of the biosensing strategy

**Figure 1:**
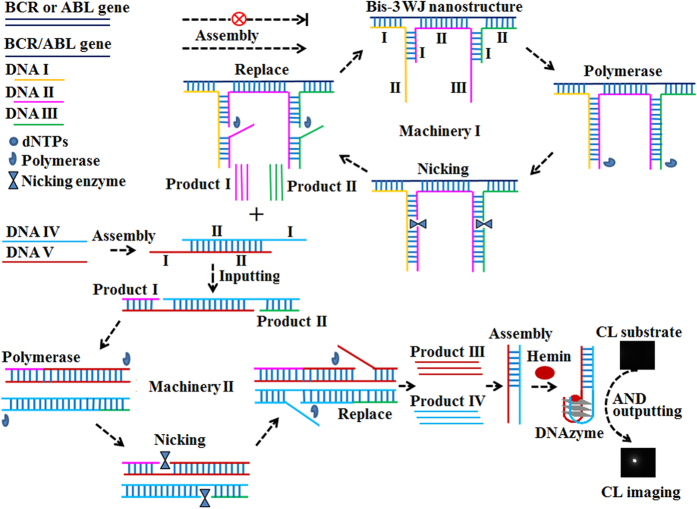
Schematic illustration of the imaging method for BCR/ABL fusion gene detection base on bis-3WJ nanostructure and DNA machineries.

The principle of CL imaging method for BCR/ABL fusion gene detection based on bis-3WJ nanostructure and DNA machineries is illustrated in Fig. 1. The system includes three tailored DNA probes (DNA I, II, III) for sensing target and forming appropriate nanostructure, and other two tailored DNA probes (DNA IV, V) for producing DNAzyme subunits. In detail, DNA I incorporates two regions, a recognition region (region I) for sensing ABL gene, and a scaffold region (region II) for producing product I. DNA II is designed with three regions: region I, region II, region III. Region I is used as primer to produce product I. Region II with eight base pairs is used for sensing ABL gene and BCR gene, respectively. Region III is used as scaffold for producing product II. DNA III includes region I as primer for producing product II and region II for recognizing BCR gene. Meanwhile, DNA VI and V are devised with the recognition region I for the respective products I and II, and scaffold region II for producing DNAzyme subunits that are complementary to each other (products III and IV). In the presence of BCR/ABL fusion gene, recognition event of DNA I, II, III to BCR/ABL fusion gene results in the formation of bis-3WJ nanostructure to activate the machinery I, leading to the autonomous replication/nicking processes and the generation of products I and II. In the cascade form, products I and II further activate machinery II by employing DNA IV and V as scaffolds, generating products III and IV. Products III and IV can synergistically form the DNAzyme nanostructure with hemin, catalyzing CL substrate for signal output. On the contrary, in the presence of one input (normal BCR gene or ABL gene) or in the absence of target, bis-3WJ nanostructure can’t be formed and cascade DNA machineries fail to be activated, leading to no signal output. Thus, only in the presence of BCR/ABL fusion gene, CL signal can be generated, resulting in an AND gate operation. Based on bis-3WJ nanostructure and cascade DNA machineries, the developed imaging strategy attains an AND logic gate operation, and ultrasensitive and specific detection of BCR/ABL fusion gene.

### Feasibility of the AND logic gate

**Figure 2:**
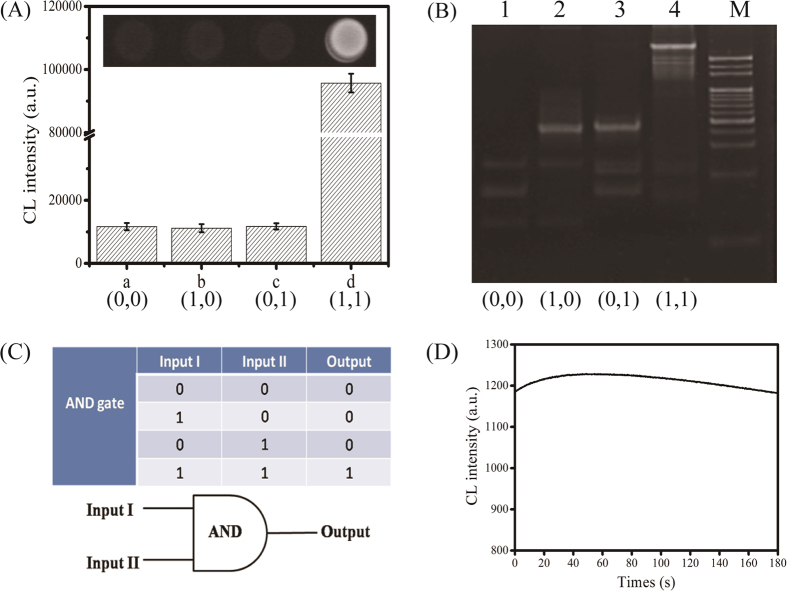
(**A**) CL images for the logic gate operation: blank (0, 0), normal ABL gene (1, 0), normal BCR gene (0, 1) and BCR/ABL fusion gene (1, 1). (**B**) Analysis on the formation of bis-3WJ nanostructure corresponding to different target inputs. (**C**) The truth table. (**D**) Analysis on the stability of CL signal.

To ensure the feasibility of AND logic gate, CL images under the action of different target inputs (100 nM) were investigated. As shown in Fig. 2A, image a represented the background signal in the absence of any target (0, 0), and images b, c and d represented the signal responses to the presence of normal ABL gene (1, 0), BCR gene (0, 1) and BCR/ABL fusion gene (1, 1), respectively. Results demonstrated only when BCR/ABL fusion gene (1, 1) was present, an enhanced CL response could be generated, leading to an AND logic gate as indicated in the truth table (Fig. 2C).

To verify whether AND logic gate was operated by target responsive bis-3WJ nanostructure as designed, gel electrophoresis analysis was performed. As shown in Fig. 2B, in the absence of any input (0, 0), the three bands corresponding to DNA I, II and III in lane 1 coexisted. The bands in lane 2 and lane 3 respectively represented the input of normal ABL gene (1, 0) and BCR gene (0, 1), resulting in no bis-3WJ nanostructure. The distinct bands with lower mobility in lane 4 represented the inputs of BCR/ABL fusion gene (1, 1), indicating the successful formation of bis-3WJ nanostructure by self-assembly. Therefore, these results demonstrated bis-3WJ nanostructure could be formed only in the presence of BCR/ABL fusion gene, further verifying the operation of AND logic gate was under the action of different target inputs (the detailed verification and gel results were shown in Fig. S2). Additionally, Fig. 2D showed the CL intensity increased quickly to the maximum value and maintained the signals above 80% of the maximum value for 3 min after adding CL substrate to reaction system, indicating the good stability of CL signals.

### Verification of cascade DNA machineries

**Figure 3:**
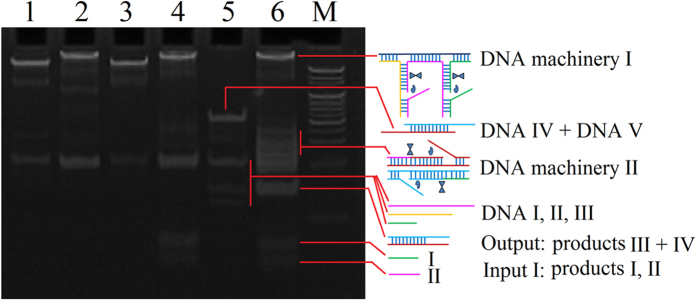
(**A**) Verification of cascade DNA machineries.

To investigate whether polymerase/nicking enzyme machineries worked as designed, a native polyacrylamide gel electrophoresis was performed (Fig. 3). The distinct band with slow migration represented bis-3WJ nanostructure (lane 1) that was verified with agarose electrophoresis in Fig. S1. Addition of dNTPs and polymerase to the mixture in lane 1 led to a distinct band with slower migration compared with lane 1, certificating polymerase process proceeded as designed (lane 2), whereas addition of dNTPs and nicking enzyme to the same mixture displayed analogical bands at the identical positions in lane 1, suggesting nicking process could not occur before polymerization (lane 3). However, addition of dNTPs, polymerase and nicking enzyme to the mixture of lane 1 resulted in two new bands (lane 4) corresponding to products I and II, indicating the polymerase/nicking enzyme machinery I worked as design. In the absence of BCR/ABL fusion gene, DNA I, II, III and the hybridization strands of DNA IV and V could coexist stably (lane 5). However, addition of BCR/ABL fusion gene, polymerase and nicking enzyme to the mixture of lane 5 (lane 6) resulted in the partly disappearance of bands of products I and II, and the appearance of hybridization brand of products III and IV, demonstrating the cascade DNA machineries worked as expected.

### Optimization of the experimental conditions

**Figure 4:**
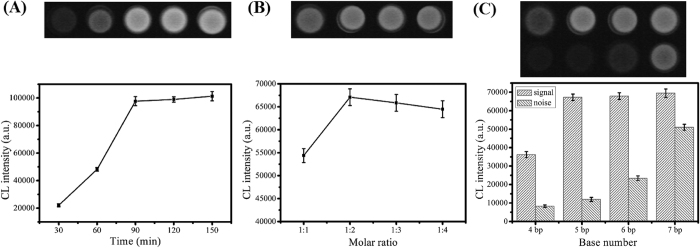
Optimization of the experimental conditions: (**A**) incubation time of the amplification reaction, (**B**) molar ratio of DNA I, II, III to DNA IV, V, (**C**) 3′ length of bis-3 WJ primer complementary to bis-3 WJ template. Error bar represents the standard deviation (n = 3).

To obtain optimum analytical performance, several experimental conditions were optimized, including the time of replication/nicking process, the molar ratio of DNA I, II and III to DNA VI and V, and the 3′ length of bis-3 WJ primer complementary to bis-3 WJ template. As depicted in Fig. 4A, with the replication/nicking process prolonged from 30 min to150 min, the CL intensity rose accordingly in the presence of target (100 nM), yet the signal started to plateau longer than 90 min. The enhanced signal within 90 min validated the feasibility of the amplification process, while the plateau phenomenon could be due to the exhaustation of almost dNTPs in the reaction solution. Therefore, the reaction time of 90 min was used for the following experiment. Subsequently, the optimum molar ratio was investigated under the ratio of 1:1, 1:2, 1:3 and 1:4 in the presence of target (1 nM). Results in Fig. 4B demonstrated that the CL response increased in the range of 1:1 to 1:2, decreased thereafter. Machinery II employed DNA IV and V as templates, and machinery I used DNA I, II and III to generate products I and II as primers of machinery II. The appropriate molar ratio of DNA I, II and III to DNA VI and V was helpful for the cascade amplification process of machinery I and II. Thus, the optimum molar ratio was selected at 1:2. Furthermore, four different bis-3 WJ primers, each having 4, 5, 6, and 7 bp complementary to bis-3 WJ templates, were evaluated. As expected, a increased base number of bis-3 WJ primers to bis-3 WJ primers led to a higher CL signal in the presence of target (1 nM) (Fig. 4C). However, a relatively strong CL intensity of background was also observed as this base number increased. The high background signal could be ascribed to the formation of a transient duplex hybrid between bis-3 WJ primers and bis-3 WJ templates. Furthermore, DNA polymerase recognized this transient duplex and then performed the cascade amplification. Therefore, to prevent the non-specific background and further improve the sensitivity of the amplification assay, 5 bp was chosen for the following detection according to the results shown in Fig. 4C.

### Sensitivity of the biosensing system

**Figure 5:**
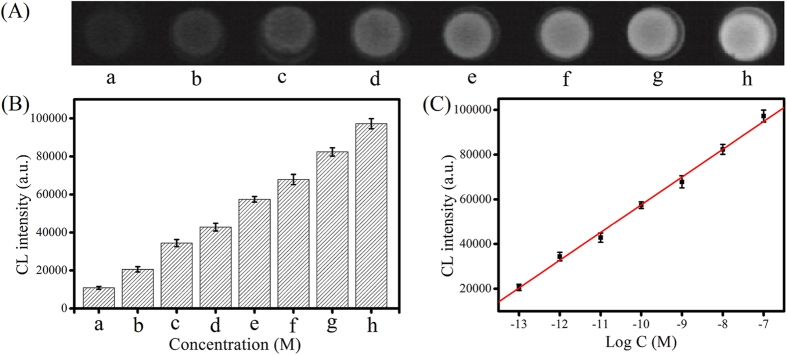
(**A**) CL images upon different concentrations of the analytes: (a) 0, (b) 1 × 10^−13^, (c) 1 × 10^−12^, (d) 1 × 10^−11^, (e) 1 × 10^−10^, (f) 1 × 10^−9^, (g) 1 × 10^−8^, (h) 1 × 10^−7^. (**B**) CL intensities corresponding to CL images. (**C**) Logarithmic plot of CL intensities versus different concentrations of targets. Error bar represents the standard deviation (n = 3).

Under optimized experimental conditions, different concentrations of BCR/ABL fusion gene were added to the designed system. The CL responses to relevant concentrations of BCR/ABL fusion gene were shown in Fig. 5A,B. As expected, a gradual increase of CL intensity was observed with the increase of BCR/ABL fusion gene concentrations. Figure 5C depicted the CL intensity was linearly dependent on the logarithm of BCR/ABL fusion gene concentration ranging from 100 fM to 100 nM, covering seven dynamic ranges (I = 181702 + 12410 log C, R^2^ = 0.9978). Based on 3 times the standard deviation over the blank response, the detection limit was estimated to be 23 fM, which is superior to those of most reported sensing method (Table S2). The excellent analytical performance could be attributed to the high amplification efficiency of cascade DNA machineries and the DNAzyme catalyzed cascade chemiluminescence. In addition, the devise of duplex templates that assembled by DNA IV and V, eliminated the destructive hybridization of free templates with the generated DNAzyme subunits^37^, enhancing the amplification efficiency.

### Specificity of the biosensing system

**Figure 6:**
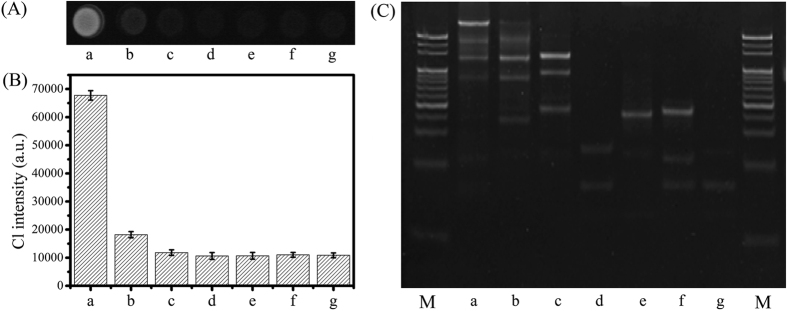
(**A**) CL responses to different targets: BCR/ABL fusion gene (a), single-base mismatched target (b), four-base mismatched target (c), non-complementary target (d), normal ABL gene (e), normal BCR gene (f) and blank control (g). (**B**) CL intensities corresponding to CL images. (**C**) Analysis on the formation of bis-3WJ nanostructure corresponding to different targets. Error bar represents the standard deviation (n = 3).

To investigate the specificity of the biosensing system, different target DNA sequences (1 nM) were tested, including perfect target (a), single-base (M1) mismatched target (b), four-base (M4) mismatched target (c), non-complementary (NC) target (d), normal ABL gene (e) and normal BCR gene (f). As shown in Fig. 6A,B, M4 target, NC target, normal ABL gene and normal BCR gene led to almost identical responses to blank control (c, d, e, f vs. g), whereas perfect target resulted in significantly increased response (a vs. g), which could be easily discriminated from the M1 target (a vs. b), indicating high specificity of the biosensing system. Furthermore, gel electrophoresis analysis was also applied to investigate the formation of bis-3WJ nanostructure that responded to different targets. As shown in Fig. 6C, BCR/ABL fusion gene resulted in a distinct band corresponding to the bis-3WJ nanostructure, which could be distinctly differentiated from the band triggered by M1 target (b), whereas M4 target, NC target (d), normal ABL gene (e), normal BCR gene (f) and the blank control (g) failed to cause the formation of the bis-3WJ nanostructure. Therefore, only the input of BCR/ABL fusion gene could specifically lead to the formation of bis-3WJ nanostructure, further implying the high specificity of the developed biosensor. The high specificity could be attributed to the precise control of the logic gate.

### Interfering effects of complex matrix

To investigate the interfering effects of biological samples on the developed chemiluminescence biosensor, the spiked assay was performed using10-fold diluted normal human serum samples as blank matrix, preliminarily. Results showed the recoveries were between 96.0% and 98.2% with the relative standard deviation (RSD) between 1.3% and 2.1% (n = 5) (Table S3), suggesting the acceptable analytical capability of the established biosensor in complex matrix.

## Conclusions

To summarize, the present study has developed an ultrasensitive and specific CL imaging method for BCR/ABL fusion gene detection based on bis-3WJ nanostructure and cascade replication/nicking machineries. The achieved ultrasensitivity of the biosensing system could be attributed to the high amplification efficiency of cascade DNA machineries and the cascade DNAzyme catalyzed chemiluminescence. And the specificity could be attributed to the precise control of the logic gate. Importantly, this imaging strategy has the advantages of isothermal, homogeneous and label-free assay system, without requirements of sophisticated operation and expensive instruments. Thus, this biosensing strategy might provide a promising alternative tool for CML diagnosis on CL imaging platform.

## Experimental

### Materials and apparatus

HPLC-purified oligonucleotides used in the experiment were synthesized by Sangon Inc (Shanghai, China), and all sequences are listed in Table S1. The Klenow fragment (exo-) DNA polymerase, Nb.BbvCI nicking endonuclease, and deoxyribonucleoside triphosphates (dNTPs) mix were purchased from New England Biolabs Ltd. (Beijing, China). Hemin was obtained from Sigma-Aldrich (St Louis, MO, USA), 4-(2-hydroxyethyl) piperazine-1-ethanesulfonic acid sodium salt (HEPES) and dimethyl sulfoxide (DMSO) were from Sangon Inc (Shanghai, China). The CL reagent kits were ordered from Advansta (California, USA). All solutions were prepared and diluted by diethyprocarbonated (DEPC)-treated water. All other reagents were of analytical grade, and Millipore-Q water (≥18 МΩ) was used in all experiments. IFFM-E luminescent analyzer (Remax, China) was used to record kinetic behavior of the CL reaction. Cool ImagerTM (Viagene Biotech Inc, USA) was implemented to capture CL images. DYY-6C electrophoresis analyzer (Liuyi Instrument Company, China) and a Bio-rad ChemDoc XRS (Bio-Rad, USA) were used for gel electrophoresis.

### Cascade amplification reaction

The amplification reaction was carried out in 20 μL of total reaction volume, consisting of 2.5 U DNA polymerase, 5 U nicking endonuclease, 500 μM dNTPs, 100 nM DNA I, II and III, 200 nM DNA VI and V, and different concentrations of BCR/ABL fusion gene. The one-pot reaction system was incubated at 37 °C for 1.5 h in a mixed buffer of NEBuffer 2 (50 mM NaCl, 10 mM Tris-HCl, 10 mM MgCl_2_, 1 mM DTT, pH 7.9) and CutSmart^®^ Buffer (50 mM potassium acetate, 20 mM tris-acetate, 10 mM magnesium acetate, 100 μg·mL^−1^ BSA, pH 7.9), followed by incubation at 80 °C for 20 min to terminate the reaction.

### Chemiluminescence measurement

The chemiluminescence system contained 12 μL of above resulting solution and 108 μL of HEPES solution (pH 8.6, 25 mM HEPES, 200 mM NaCl, 20 mM KCl, Triton X-100 (0.05%, w/v), and DMSO (1%, v/v)). Then, 3 μL of 10 μM hemin solution was added to the system, followed by incubation for 30 min at room temperature. After that, 16 μL of CL substrate was transferred to the system, and CL signals were collected by CCD with a 3 min dynamic integration.

### Native polyacrylamide gel electrophoresis

Native polyacrylamide gel electrophoresis (PAGE) was performed on 10% acrylamidein 1×TBE buffer (90 mM Tris-HCl, 90 mM boric acid, 2 mM EDTA, pH 7.9) at constant voltage of 148 V for 35 min, and agarose gel electrophoresis was performed on 2.5% agarose for 25 min at 120 V. The gels were imaged using a Molecular Imager Gel Doc XR (Bio-Rad, USA).

## Additional Information

**How to cite this article**: Xu, Y. *et al*. Bis-three-way junction nanostructure and DNA machineries for ultrasensitive and specific detection of BCR/ABL fusion gene by chemiluminescence imaging. *Sci. Rep.* **6**, 32370; doi: 10.1038/srep32370 (2016).

## Supplementary Material

Supplementary Information
