# Supplementary material for: Bis-three-way junction nanostructure and DNA machineries for ultrasensitive and specific detection of BCR/ABL fusion gene by chemiluminescence imaging
Source: Sci Rep. 2016 Aug 31;6:32370. doi: 10.1038/srep32370 (PMC5006031; doi:10.1038/srep32370)
Supplement: Supplementary Information [file srep32370-s1.docx]

**Supplementary Information**

**Bis-three-way junction nanostructure and DNA machineries for ultrasensitive and specific detection of BCR/ABL fusion gene by chemiluminescence imaging**

Authors:

Yongjie Xu^1,*^, Xintong Bian^1,*^, Ye Sang^1^, Yujian Li^1^, Dandan Li^1^, Wei Cheng^3^, Yibing Yin^1^, Huangxian Ju^1,2^ & Shijia Ding^1^

Author affiliations:

^1^Key Laboratory of Clinical Laboratory Diagnostics (Ministry of Education), College of Laboratory Medicine, Chongqing Medical University, Chongqing 400016, China. ^2^State Key Laboratory of Analytical Chemistry for Life Science, Department of Chemistry, Nanjing University, Nanjing 210023, China. ^3^The Center for Clinical Molecular Medical detection, The First Affiliated Hospital of Chongqing Medical University, Chongqing 400016, China.

Corresponding author:

Shijia Ding, College of Laboratory Medicine, Chongqing Medical University, Chongqing, 400016, China. Fax: +86 23 68485786; Tel: +86 23 68485688; E-mail address: [dingshijia@163.com](mailto:dingshijia@163.com) (S.J. Ding).


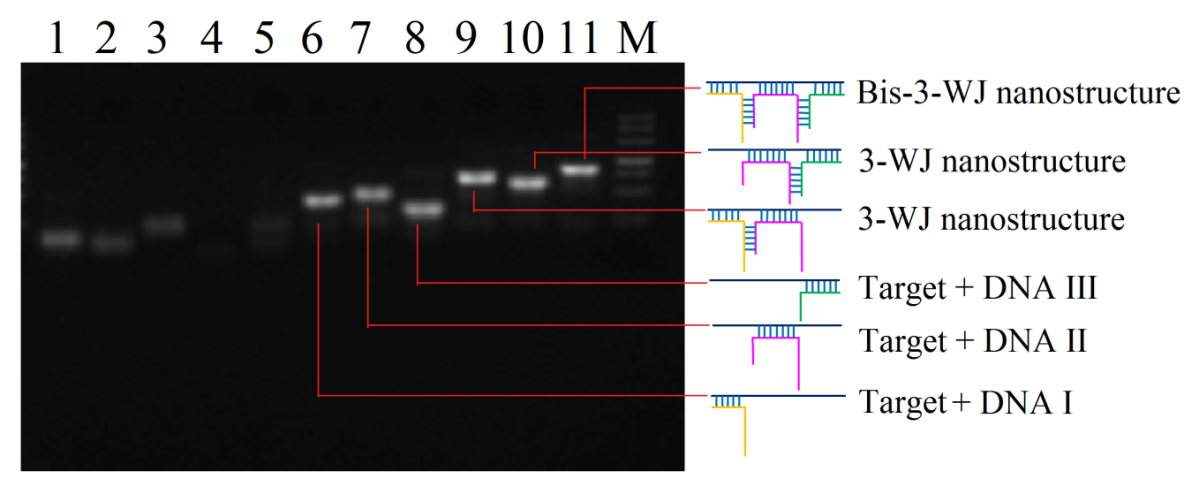


**Figure S1** Verification of the bis-3WJ nanostructure

An agarose gel electrophoresis was performed to verify the self-assembly of bis-3WJ nanostructure corresponding to BCR/ABL fusion gene. The bands in lane 1, lane 2, lane 3 and lane 4 represented BCR/ABL fusion gene, DNA I, II and III, respectively. And the bands in lane 5 represented the mixture of DNA I, II and III. The distinct bands in lane 6, lane 7 and lane 8 with lower mobility corresponded to the hybridization of BCR/ABL fusion gene with DNA I, II and III, respectively. The distinct bands in lane 9 and lane 10 with even lower mobility respectively represented one 3-WJ nanostructure, each consisting of BCR/ABL fusion gene, DNA I, II and BCR/ABL fusion gene, DNA II, III. The most distinct bands in lane 11 with lowest mobility demonstrated the formation of bis-3WJ nanostructure assembled by BCR/ABL fusion gene, DNA I, II and III.

**
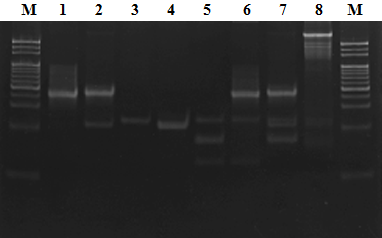
**

**Figure S2** Verification of the AND logic gate

Native polyacrylamide gel electrophoresis (PAGE) was performed on 10% acrylamidein to verify whether the formation of bis-3WJ nanostructure was corresponded to different target as the truth table. The bands in lane 1, lane 2, lane 3, lane 4, lane 5, lane 6, lane 7, lane 8 and lane M represented hybridization products of ABL gene with DNA I and BCR gene with DNA III, normal ABL gene, normal BCR gene, the mixtures of DNA I, II and III (0, 0), ABL gene with DNA I, II, III (1, 0), BCR gene with DNA I, II and III (0, 1), BCR/ABL fusion gene with DNA I, II, III (1, 1) and 20 bp DNA ladder. As shown in Figure S2, when compared with the bands in lane 5, disappear of DNA I in lane 6 indicated the hybridization of ABL gene and DNA I, while disappear of the shortest DNA III in lane 7 demonstrated the hybridization of BCR gene and DNA III. In addition, to further characterize the assembly, we added another two lanes to verify the assembly of ABL gene with DNA I (lane 1), and BCR gene with DNA III (lane 2). Gel results showed the bands with lowest mobility in lane 6 and lane 7 were at identical positions with lane 1 and lane 2, respectively, indicating ABL gene and BCR gene have no unexpected interaction with another two designed DNA. Therefore, the assembly that triggered by different targets was worked as the truth table.

**Table S1** DNA sequences employed in this work

| Oligonucleotides | Sequences (5'-3') |
| --- | --- |
| DNA I | GTTGTACACTTGCCTCAGCGAGAACGTTCAGCGGCCAGTAGCATCT  Region II Region I |
| DNA II-4 | CATCACTCTTCACCTCAGCCAGTCTCTTGAGTTCAAAAGCCCTTTTCGTT |
| DNA II-5 | CATCACTCTTCACCTCAGCCAGTCTCTTGAGTTCAAAAGCCCTTTTCGTTC  Region III Region II Region I |
| DNA II-6 | CATCACTCTTCACCTCAGCCAGTCTCTTGAGTTCAAAAGCCCTTTTCGTTCT |
| DNA II-7 | CATCACTCTTCACCTCAGCCAGTCTCTTGAGTTCAAAAGCCCTTTTCGTTCTC |
| DNA III-4 | CCACTCAGCCACTGGATTTAAGCATTGAGA |
| DNA III-5 | CCACTCAGCCACTGGATTTAAGCATTGAGAC  Region II Region I |
| DNA III-6 | CCACTCAGCCACTGGATTTAAGCATTGAGACT |
| DNA III-7 | CCACTCAGCCACTGGATTTAAGCATTGAGACTG |
| DNA IV | TGTCGAGAATGCTAGAA**CCCAA**CCCTCAGCGTTGTACACTTGCCT  Region II Region I |
| DNA V | CCCTACCCATCTAGCATTCTCGACACCTCAGCATCACTCTTCACCT  Region II Region I |
| BCR/ABL | CAGATGCTACTGGCCGCTGAAGGGCTTTTGAACTCTGCTTAAATCCAGTGGCTGAGTGG |
| ABL | CAGATGCTACTGGCCGCTGAAGGGCTTCTGGAAGAGAAAGGGGGGAACAGAAAAAAGAA |
| BCR | AACCCTCCTCCCCAAACCAGTACTTACTTGAACTCTGCTTAAATCCAGTGGCTGAGTGG |
| M1 | CAGATGCTACTGGCCGCTGAAGGGCATTTGAACTCTGCTTAAATCCAGTGGCTGAGTGG |
| M4 | CAGATGCTACTGGCCGCTGAACGGCTTAAGAACCCTGCTTAAATCCAGTGGCTGAGTGG |
| NC | GACACCAGAAGCAGCAACAACGATTGTTTCGCCAATGAAGACATATTCTTCTGCGCCAG |

The underline portion represents mutation bases in BCR/ABL fusion gene. M1 Single-base mismatched target, M4 four-base mismatched target, NC Non-complementary target. Green represents nicking site sequence, Red represents the 3′ length of bis-3 WJ primer complementary to bis-3 WJ template.

**Table S2** Comparisons between the developed imaging method and other reported methods for the detection of BCR/ABL fusion gene

| Detection method | LOD | Reference |
| --- | --- | --- |
| Electrochemical | 2.56 pM | S1 |
| Electrochemical | 1.05 pM | S2 |
| Electrochemical | 83 fM | S3 |
| Electrochemical | 6.7 nM | S4 |
| Electrochemical | 2.11 pM | S5 |
| Chemiluminescence | 23 fM | this work |

**Table S3** The recoveries determined using the developed method via spiking synthetic DNA into 10-fold diluted human normal serum samples

| Sample no. | Spiking value | Assayed value | Recovery | RSD |
| --- | --- | --- | --- | --- |
| 1 | 0.50 pM | 0.48 pM | 96.0% | 2.1% |
| 2 | 5.00 pM | 4.91 pM | 98.2% | 1.3% |
| 3 | 50.00 pM | 48.65 pM | 97.3% | 1.4% |

**References**

1. Sharma, A., *et al*. Chitosan encapsulated quantum dots platform for leukemia detection. *Biosens. Bioelectron*. 38, 107-113 (2012).
2. Chen, X., *et al*. Coupling a universal DNA circuit with graphene sheets/polyaniline/AuNPs nanocomposites for the detection of BCR/ABL fusion gene. *Anal. Chim. Acta* 889, 90-97 (2015).
3. Lee, A.C., *et al*. Electrochemical detection of leukemia oncogenes using enzyme-loaded carbon nanotube labels. *Analyst* 139, 4223-4230 (2014).
4. Chen, J., Zhang J., Huang L., Lin X. & Chen G. Hybridization biosensor using 2-nitroacridone as electrochemical indicator for detection of short DNA species of Chronic Myelogenous Leukemia. *Biosens. Bioelectron*. 24, 349-355 (2008).
5. Wang, L., *et al*. Graphene sheets, polyaniline and AuNPs based DNA sensor for electrochemical determination of BCR/ABL fusion gene with functional hairpin probe, *Biosens. Bioelectron*. 51, 201-207 (2014).
